# Urban malaria and associated risk factors in Jimma town, south-west Ethiopia

**DOI:** 10.1186/1475-2875-10-173

**Published:** 2011-06-24

**Authors:** Abebe Alemu, Wondewosen Tsegaye, Lemu Golassa, Gemeda Abebe

**Affiliations:** 1 Department of Medical Laboratory Sciences and Pathology, Jimma University, Jimma, Ethiopia; 2 Department of Medical Laboratory Sciences, College of Medicine and Health Sciences, University of Gondar, Gondar, Ethiopia 6543; 3 Department of Epidemiology and Social Medicine, University of Antwerp, Antwerp, Belgium

## Abstract

**Background:**

Malaria kills millions around the world. Until recently it was believed to be a disease of rural areas, since the *Anopheles *mosquito, which transmits *Plasmodium *species breeds in rural areas. Urban malaria is emerging as a potential, but "avertable" crisis, in Africa. In view of the rapidly growing number of small and medium-sized towns in Ethiopia there is a pressing need to improve the understanding of the epidemiology of malaria. Therefore, the aim of this study was to determine malaria prevalence and associated risk factors in Jimma town.

**Methods:**

A cross-sectional study was carried out in Jimma town from April 1 to May 28, 2010. 804 study participants were included from 291 households for microscopic examination of malaria parasites. Socio-demography data and risk factors were collected using structured questionnaires. Logistic regression analysis was done using SPSS 15.0 statistical software.

**Results:**

From a total of 804 study participants in current survey only 42 (5.2%) were positive for malaria parasites. *Plasmodium vivax, Plasmodium falciparum *and mixed infection accounted 71.4%, 26.2% and 2.4%, respectively. Higher malaria prevalence rate was observed among under-five children (11%). Those who do not use insecticide-treated bed nets (ITN) were more likely to be infected with malaria (OR = 13.6; 95% CI 4.9-37.2, p < 0.001) compared with those who use the ITN. Living in areas where stagnant water existed (OR = 2.1; 95% CI 1.00-4.2, p = 0.047) and its distance of existence <1 km from the house(OR = 2.1; 95% CI 2.0-15.8, p = 0.001) were more likely to be infected with malaria parasite compared with those who live away from stagnant at a distance greater than 1 km.

**Conclusion:**

Malaria is a major health problem with *P. vivax *becoming a predominant species in the town. The prevalence was strongly associated with proximity of residence to potential mosquito breeding sites. Malaria is affecting significant proportions of the urban settlers and human activities nevertheless play an important role in bringing the mosquito breeding sites closer to residences.

## Background

Malaria can no longer be considered as just a rural issue in Africa where a significant and increasing proportion of the African population lives in urban areas and malaria transmission in urban settings, albeit lower level than rural areas [1-3]. By virtue of the unprecedented urbanization in Africa the scale and impact of urban malaria is increasing, Moreover, this urbanization often results in profound demographic, ecological, and socio-economic changes that are characterized by a high degree of spatial and temporal heterogeneity [4-8]. The adaptation of malaria vectors to urban ecosystems has been documented and warrants close attention [9].

Despite progress in fighting malaria worldwide, the parasitic disease kills close to 800,000 People annually. Children less than five years of age living in sub-Saharan Africa are mainly the affected groups. The disease accounts for an estimated loss of 44.7 million disability adjusted life years (DALYs), more than 80% of which are currently concentrated in sub-Saharan Africa [10]. As a matter of facts, malaria prevalence is highest among the poorest sections of the society, since they cannot afford protection from malaria through improved housing, clean environment and are particularly vulnerable to the impact of ineffective diagnosis and treatment [3].

Malaria is a leading public health problem in Ethiopia where an estimated 68% of the population lives in malarious areas and ¾ of the total land mass is regarded as malarious [11]. *Plasmodium falciparum *and *Plasmodium vivax *are the two predominant malaria parasites, accounting for 60% and 40% of malaria cases, respectively [12]. Data from health institutions indicate that clinical malaria accounts for 10-40% of all out patient consultations, with corresponding proportional morbidity among children under five years in age being 10-20% (Ghebreyesus TA, Deressa W, Witten KH, Getachew A, Sobixa T: **Malaria; The ecology and epidemiology of health and diseases in Ethiopia**, submitted).

In Ethiopia, the prevention and control activities of malaria as guided by the National Strategic Plan (2006-2010) mainly focus on rural areas [13,14]. This is because until recently, it was presumed that urban development was generally believed to reduce the risk of vector breeding, and thus malaria transmission. However, many African countries including Ethiopia have declining economies, and most cities are struggling to cope with the pace and the extent of urbanization. Indeed, in most urban areas of the developing countries, poor housing, lack of sanitation and drainage of surface water could likely increase vector breeding and human vector contact, and thus pose exclusive challenges to malaria control [15-17].

Since malaria transmission in urban settings is usually lower and more focal than in rural settings [2], urban areas hold promise for vector control and integrated vector management [18]. Again rapid urbanization alters the frequency and transmission dynamics of malaria, with significant effects on disease associated morbidity and mortality, which in turn has important implications for control [16]. Despite these realities, most of the previous researches pertaining to urban malaria in sub-Saharan Africa have been focusing on large cities [19,20]. On the other hand, the preceding malaria researches in Ethiopia essentially focused in rural areas and as a result few researches have been addressing urban malaria [21-28].

In view of the rapidly growing number of small and medium-sized towns in Ethiopia, there is a pressing need to enhance our understanding of malaria epidemiology in those settings. Like most towns of developing countries, in Ethiopia towns are also characterized by poor housing, lack of proper sanitation, poor drainage of surface water, weak health services and wide spread economic disparity, which independently or together pave the way for urban malaria transmission [23]. In order to design and implement cost-effective appropriate interventions, knowledge on local prevalence, distribution malaria and its influencing factors are nevertheless paramount importance. Therefore, this study was initiated as to assess prevalence of malaria and its predisposing factors in Jimma town.

## Methods

### Study area

**Figure 1:**
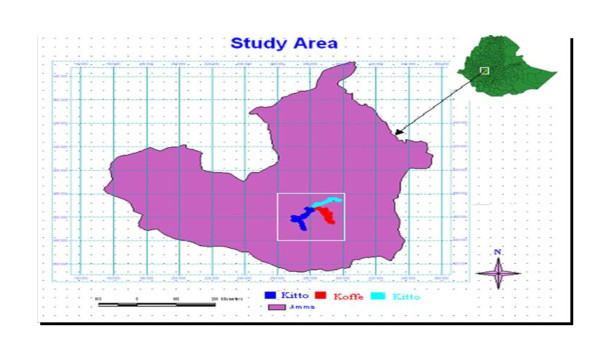
**Map of the study area**. The figure shows that Ethiopia and study area, Jimma town where Parasitological survey took place for this study.

The study was conducted at Jimma town, located 350 km south-west of Addis Ababa. The town's geographical coordinates are 7°41' N latitude and 36° 50' E longitude. The town is found at an average altitude of about 1,780 m above sea level. It lies in the climatic zone locally known as "Woyna Daga" (1,500-2,400 m above sea level) which is considered ideal for agriculture as well as human settlement. The town is generally characterised by warm climate with a mean annual maximum temperature of 30°C and a mean annual minimum temperature of 14°C. The annual rainfall ranges from 1138-1690 mm. The maximum precipitation occurs during the three months period from June through August, with minimum rainfall occurring in December and January. From a climatic point of view, abundant rainfall makes this region one of the best watered of Ethiopian highland areas, conducive for agricultural production (Figure 1).

### Sampling techniques

The survey was conducted in four Kebeles (smallest administrative units) randomly selected from all kebeles in the town assuming that the Kebeles are homogen. The number of households to be selected from four Kebeles was determined by dividing the sample size to the average number of family size per household (Statistical Report of Population and Housing Census of Ethiopia, 2007). The list of households for the selected Kebeles, knowing that the list did not contain any hidden order was obtained from the Kebele leaders and it was used as a sampling frame. Simple random sampling method was employed to select households from each Kebeles from household registry using a table of random numbers and when the selected household was inconvenient, the households before or after the indicated one was sampled for replacement. All individuals who were members of randomly selected households and who slept there In the previous night were included in the study and any relatives who come to those households during study and any individuals who were not willing to participate in the study were excluded from the study.

### Sample size and sample selection

The sample size for the study was estimated using the formula for estimating single proportion at 95% confidence interval (CI) level (Z (1-ά/2) = 1.96), an expected prevalence of 10.3% reported by Yewhalaw (2010) [27] around Jimma, 2.0% margin of error, and 5% level of significance. A sample of 804 individuals (~291 households) was estimated as the minimum number required for malaria parasite prevalence testing in the town.

### Parasitological survey

The staining techniques and blood film examination for malaria parasite detection was conducted according to a standard operating procedure (SOP) in Jimma University specialised hospital laboratory. Briefly, peripheral blood was collect from finger by disposable blood lancet and thick and thin films were made on the same slide. After being air-dried in a horizontal position, the thin blood films were fixed in methanol for 30 sec. Then smears were stained with 10% Giemsa solution for 20 min. Each slide was examined under oil immersion microscopic objective by experienced laboratory technicians who were certified on malaria diagnosis and species identification from Ethiopian Ministry of Health. Hundred fields were examined before negative result was reported. The thin smear was used to identify the type of *Plasmodium *species. For quality control each slide were examined twice by two different laboratory technicians. The second round confirmatory microscopic examination done by experienced laboratory technician who was blind for the first result.

### Data analysis

After data collection process, the data were checked for completeness and any incomplete or misfiled questionnaires were sent back to the respective data collector for correction. The laboratory investigation result recording format for each participant were carefully filled and attached with the respective questionnaire. Data were double entered and analysed by using SPSS-15.0 statistical software (SPSS Inc. Chicago, 2007). Descriptive analysis was computed for both dependent and in dependent variables. The frequency distribution of both dependent and independent variables were worked out and the association between the independent and dependent variables were measured and tested using OR and 95% CI. The relative contribution of each selected variables to the outcome of interest were determined using logistic regression. The significant level was consider at P < 0.05.

### Ethical consideration

Ethical clearance was obtained from Jimma University, College of Public Health and Medical Sciences. Written consent was obtained heads of household and study participants. All cases with history of fever in the preceding three days and/or those who had fever on examination and positive for malaria parasite during blood film examination were offered anti-malarial treatment as per national guideline.

## Results

### Sociodemography characteristics of household respondents

Of a total 291 household heads, 166 (57%) interviewees were males and 125 (43%) were females. Most respondents were married (85.2%) with a median age of 40 years. Majority of the respondents (57.4%) were Oromo by ethnicity and Muslim was the predominant religion in the area accounting for 41.9%. The family size of the population ranged from 1 to 12 with an average of 4.7. About 27.5% of the study participants were illiterate while 72.5% of them were literate. Of the total respondents, 56.4% of them were engaged in private business and 44.7% of the participants were supposed to have a monthly income less than 31.25 USD (Table 1).

**Table 1:** Sociodemography characteristics of household respondents, Jimma town, 2010

Variables		Frequency	Percentage
Age	15-40	190	65.3
	41-64	85	29.2
	>64	16	5.5
Sex	Male	166	57.0
	Female	125	43.0
Religion	Muslim	122	41.9
	Catholic	5	1.7
	Orthodox	118	40.5
	Protestant	45	15.5
	Other	1	0.3
	Oromo	167	57.4
Ethnicity	Amhara	55	18.9
	Dawuro	50	17.2
	Kaffa	10	3.4
	Tigrea	9	3.1
Marital Status 1	Unmarried	17	5.8
	Married	248	85.2
	Widowed	9	3.1
	Divorced	17	5.8
Educational status	Illiterate	80	27.5
	Read and write	6	2.1
	Grade1-4	19	6.5
	Grade 5-8	92	31.6
	High school & above	94	32.3
Family size	1-5	218	74.9
	6-10	69	23.7
	>10	4	1.4
Monthly income	< 31.25USD	130	44.7
	31.25-62.5USD	104	35.7
	>62.5USD	57	19.6
Occupation	Private business	164	56.4
	GO employed	64	22.0
	House wife	32	11.0
	Daily laborer	30	10.3
	NGO worker	1	0.3
	private business	164	56.4
No of bed room	One bed room	163	56.0
	Two bed rooms	95	32.6
	Three bed rooms	19	6.5
	More than three	14	4.8
Type of house	Mud plastered	243	83.5
	Stone wall	41	14.1
	Break walls	7	2.1
			

Note, NGO = nongovernmental organization, GO = governmental organization

### Knowledge, attitude and practice of household respondents

Knowledge, attitude and practice (KAP) of household respondents for malaria were presented below (Table 2). Most respondents (71.8%) replied that *Plasmodium *is the causative agent of malaria and others reported that unhygienic condition (13.1%) and cold weather (1.4%) could cause malaria and small number of respondents (13.8%) did not know the causative agent of malaria. Two-third of the respondents (67.4%) replied that malaria is a transmittable disease and more than one out of five (21.3%) did not know whether or not malaria is transmittable disease while 11.3% of them believe that malaria is none transmittable disease (Table 2).

**Table 2:** Knowledge, attitude and practice of household respondents towards malaria, Jimma town, 2010

Variables		Household respondents (N = 291)	
		
		Frequency	Percentage
Cause of malaria	*Plasmodium *	209	71.8
	Unhygienic condition	38	13.1
	Cold weather	4	1.4
	Not know	40	13.8
Malaria is transmittable	Yes	196	67.4
	No	33	11.3
	Not know	62	21.3
Ways of malaria transmission	Mosquito bite	173	59.5
	Body contact with infected persons	16	5.5
	Respiratory routes	2	0.7
	Not know	100	34.4
Breeding site of mosquito	Stagnant water	228	78.4
	Animal wastes	3	1.0
	Not know	60	20.6
Malaria is treatable and preventable	Yes	256	88
	No	6	2
	Not know	29	10
Outcome of malaria if not treated early	Death	211	72.5
	Self-cure	46	15.8
	Disability	2	0.6
	Not know	32	11.1

### Health service and environmental factors

Distribution of some health service and environmental factors were presented in Table 3. Of the total 291 household heads interviewed, 84% replied that they had experienced malaria and had used anti-malaria drugs. With regard to the brand of anti-malaria drugs they used, 47% replied chloroquine (CQ) and 23% Coartem^®^, whereas 28% indicated that they did not know its brand (Table 3).

**Table 3:** Distribution of respondents to some health service and environmental factors, Jimma town, 2010

Variables		Household respondents (N = 291)	
		
		Frequency	Percentage
Previous malaria history and who got treatment	Yes	244	84
	No	47	16
Type of malaria drug used	Chloroquine	115	47
	Coartem	56	23
	Other antimalarial drug	73	30
Availability of ITNs	One ITN	87	29.9
	More than ITNs	138	47.8
	No	66	22.3
Usage of ITNs in the home	Yes	145	64.4
	No	80	35.6
Family members who use ITNs	Whole family	11	7.6
	Some family members	134	92.4
Presence of stagnant water	Yes	167	57.4
	No	124	42.6
Distance stagnant water nearby the home	<1 km	89	53.3
	≥1 km	78	46.7
Chemical spraying habit for mosquito control	Yes	62	21.3
	No	229	78.7

### Malaria parasite prevalence

A total of 804 individuals were enrolled in the study out of 291 households from four kebeles (Bosa Kito, Ginjo Guduru, Seto Semero and Bacho Bore) in Jimma town during the minor malaria transmission season (April to May, 2010). The study population was composed of individuals aged and above 15 years (54.5%), followed by 5-14 years age groups (36.6%) and 9.0% aged below 5 years with a median age of 21 (± 1.2sd) years. Of the total study population, 469 (58.3%) were females and 335 (41.7%) were males with a sex ratio of 1:1.4. The prevalence of malaria was 5.2% (n = 804) in the study area where *P. vivax *accounted for 30 (71.4%), and *P. falciparum *for 11 (26.2%), while mixed species infection (Pf & Pv) accounted for 2.4% (1/804).

Of the total *Plasmodium *infected subjects, 4.76% were found at the gametocyte stage of *P. falciparum *and the remaining 88.2 and 7.1% of them were at their early stage (ring/trophozoite stage) and mature schizont stages of both malaria parasites and *P. vivax *respectively.

### Univariate risk factor analysis for plasmodium infection in study participants

In univariate analyses of selected sociodemographic characteristics of the study participants, significant positive associations were observed between infection of any *Plasmodium *species in the participants and age groups where more cases were observed in age groups between 0-4 years (OR = 5.3; 95% CI 2.0-13.8, p = 0.001) and between 5-4 years (OR = 3.8; 95% CI 1.8-8.1, p = 0.001) compared to age group above 14 years. Even though there was no statistically significant difference of malaria prevalence between sex or among kebeles, the prevalence was higher in the males (6.3%) than females (4.5%) and highest prevalence of microscopically confirmed malaria cases were observed in locality (Kebele) called Bosa Kito (7%).

Although there was no overall significant difference in malaria prevalence between monthly income of the households, significant more cases of malaria were observed in those having monthly income<31.25 USD (OR = 3.7; 95% CI 1.1-12.8, p = 0.042) compared to having >62.5 USD. Other variables under analyses did not result any association (Table 4). Similarly in univariate analyses of environmental factors, significant positive associations were seen between presence of any *Plasmodium *species in the participants and none usage of ITNs in home (OR = 13.6;95% CI 4.9-37.2, p = 0.000), living in areas where stagnant water existed (OR = 2.05;95% CI1.00-4.2, p = 0.047) and its distance of existence <1 km from the house (OR = 2.1; 95% CI 2.1-15.8, p = 0.001) (Table 4).

**Table 4:** The distributions of some selected sociodemographic, health service and environmental risk factor univariate analysis for malaria, Jimma town, 2010

Variables	Microscopically confirmed malaria cases in the study subjects (N = 42)			
	
	N (%)	n(%)	Crude OR (95% CI)	P.value
Education status of HH				
Non educated	80 (27.5)	13(16.3)	0.82(0.403,1.673)	0.587
Formal education	211(72.5)	29(13.7)		
Family size				
1-5	218(74.9)	31(14.2)	1	0.837
6-10	69(23.7)	10(14.5)	1.02(0.473,2.209)	0.955
>10	4(1. 4)	1(25)	2.01(.203,19.953)	0.551
Monthly Income of HH				
< 31.25USD	130(44.6)	22(16.9)	3.67(1.051,12.794)	0.042
31.25-62.5USD	104(35.7)	17(16.3)	3.52(.984,12.568)	0.053
>62.5USD	57 (19.7)	3(5.3)	1	0.116
Age of study subjects				
0-4	73(9.1)	8(11)	5.26(2.001,13.803)	0.000
5-14	294 (36.6)	24(8.2)	3.81(1.794,8.092)	0.001
>14	437 (54.3)	10(2.3)	1	0.001
Sex of study subjects				
Male	335(41.7)	21(6.3)	1.4(0.769,2.660)	0.256
Female	468(58.3)	21(4.5)		
Kebeles of study subjects				
Bosa Kito	142(17.7)	10(7)	2.078(0.771,5.598)	0.148
Bacho Bore	302(36.6)	19(6.3)	1.841(0.759,4.465)	0.177
Ginjo Guduru	160(19.9)	6(3.8)	1.069(0.352,3.245)	0.907
Seto Semero	200(28.9)	7(3.5)	1	0.332
Availability of ITNs in the home				
No	66(22.7)	8(12.1)	.920(.378,2.239)	0.853
One ITN	87(29.9)	16(18.4)	1.502(.721,3.132)	0.277
More than one ITNs	138(47.4)	18(13)	1	0.452
Usage of ITNs in the home				
Yes	145 (64.4)	22(15.2)		
No	80 (35. 6)	20(25)	13.59(4.966,37.21)	<001
Family members who use ITNs				
Whole family	45(31)	1(2.2)		
Some family members	100(69)	11(11)	5.44(0.680,43.475)	0.110
Presence of stagnant water				
Yes	167(57.4)	30(18)	2.05(1.00,4.176)	0.047
No	124(42.6)	12(9.7)		
Distance stagnant water nearby their home				
<1 km	89 (53.3)	25(28.1)	5.70(2.062,15.771)	0.001
≥1 km	78(46.7)	5(6.4)		

Note: N = total number of study participants, n = positive for *Plasmodium *species

### Multivariate logistic regression analysis of selected variables

All sociodemographic, health service and environmental factors that showed significant associations with malaria prevalence in univariate analysis were selected and entered for multivariate logistic regression analysis to identify the most important predictors of malaria risk factors. In multivariate analysis, after controlling for kebele and monthly income, the only significant predictors of *Plasmodium *infection in study participants were existence of stagnant water (adjusted OR = 4.8; 95% CI 1.4-17.7, p = 0.015) and age groups where age groups between 0-4 years more affected compared to other age groups (adjusted OR = 5.2; 95% CI 1.5-13.8, p < 0.001) (Table 5).

**Table 5:** Multivariate logistic regression analysis of malaria prevalence with selected seemingly significant variables, Jimma town, 2010

Variables	Microscopically confirmed malaria cases in the study subjects (N = 42)					
	
	N (%)	n(%)	β	SE	Adjusted OR 95%CI	P.value
Monthly Income of HH						
< 31.25USD	130(44.7)	22(16.9)	2.106	1.058	8.212(1.033,65.30)	.047
31.25-62.5USD	104(35.7)	17(16.3)	1.027	.693	2.7(0.72,10.86)	.138
>62.5USD	57(19.6)	3(5.3)			1	
Age of study subjects						
0-4	73(9.1)	8(11)	4.3	.535	5.16(1.501,13.803)	0.000
5-14	294 (36.6)	24(8.2)	4.3	.695	3.51(1.001,8.092)	0.000
>14	437 (54.3)	10(2.3)			1	
Kebeles of study subjects						
Bosa Kito	142(17.7)	10(7)	3.97	.722	2.078(0.771,5.598)	0.148
Bacho Bore	302(36.6)	19(6.3)	3.89	.586	1.841(0.759,4.465)	0.177
Ginjo Guduru	160(19.9)	6(3.8)	.134	.638	1.069(0.352,3.245)	0.883
Seto Semero	200(28.9)	7(3.5)			1	
Availability of ITNs in the home						
No	66(22.7)	8(12.1)	-2.8	1.036	0.063(0.008,.476)	0.007
One ITN	87(29.9)	16(18.4)	-2.1	.916	.127(0.021,0.765)	.024
More than one ITNs	138(47.4)	18(13)			1	
Presence of stagnant water						
Yes	167(57.4)	30(18)	1.57	0.648	4.832(1.36,17.2)	0.015
No	124(42.6)	12(9.7)			1	

Note: N = total number of study participants, n = positive for *Plasmodium *species

## Discussion

Sub-Saharan Africa is characterized by a wave of rapid urban population increase particularly in areas where the highest rates of *P. falciparum *are common. Rapid urbanization brings about major changes in ecology, social structure and disease patterns in these countries [5]. It is estimated that 300 million people currently live in urban areas in Africa and two-thirds of them are at risk of malaria [3]. Ethiopia towns are also characterized by poor housing, lack of proper sanitation, poor drainage of surface water, weak health services and wide spread economic disparity, which independently or together facilitate urban malaria transmission [27,28].

The results of this study revealed that malaria parasite prevalence was 5.2% of which *P. vivax *accounts for 71.4%, *P. falciparum *26.2% and mixed infection only accounts 2.4%. This finding was similar with the prevalence of malaria in Gondar town, 5.3% [28] but higher than the study conducted Hawassa town, 3.9% [23]. This cross-sectional study was conducted during the minor malaria transmission season between April and May, but that of Hawassa was conducted in dry season.

In Ethiopia, epidemiological pattern of malaria transmission is generally unstable and seasonal, the level of transmission varying from place to place because of altitude and rainfall patterns. The major transmission of malaria follows the June to September major rain seasons and occurs between Septembers and December, while the minor transmission season occurs between April to May following the February to March small showers of rains. Some localities also experience perennial malaria, because the environmental and climatic situations permit the continual breeding of vectors in permanent breeding sites [24].

Seasonal variation in malaria transmission is a well-established feature of unstable malaria. In Ethiopia had reported 2.6% in dry season (April/May) and 5.8% during wet season (September-November) [26]. Similarly, malaria prevalence surveys in Kassena-Nankana district of northern Ghana showed 22% parasite rate in May (dry-low transmission) and 61% in November (wet-high transmission) [29].

This study revealed that *P. vivax *was the predominant species in the study area, unlike the previous paradigm of Plasmodium species composition in Ethiopia (*P. falciparum *60% and *P. vivax *40% of the total malaria cases) [14]. Though multitude of factors are supposed to orchestrate the shift in magnitude of the prevalence of *P. falciparum *to *P. vivax *needs, further elaborative research will be required to identify the causation and this will not be addressed in this study. This finding is in agreement with the current trends shift in malaria cases occurrence during record review from *P. falciparum *to *P. vivax*. Until 2008, the dominant species was *P. falciparum*, but since 2008 *P. vivax *was becoming the dominant malaria parasite in Ethiopia in general and in the study area in particular [30].

Several studies indicate that the use of ITNs significantly reduce the proportion of malaria morbidity and mortality [31]. To the contrary, some studies conducted in African countries revealed that the use of ITNs didn't show a significant difference in malaria morbidity and mortality [32]. A difference was observed in malaria prevalence among ITNs users and non-users in our study. But the mere presence of ITNs in households may not protect individuals from malaria morbidity unless it is properly used that could also be the implication of this finding.

Malaria is governed by a number of environmental, socio-demographic and economic factors, which affect its distribution, seasonal occurrence and transmission intensity. In contrast to other studies from the survey none of socio-demographic and economic factors had significantly associated with malaria transmission in the study area. Among the socio-demographic factors, studies indicate that malaria morbidity and mortality in individuals under 5 years of age is higher compared to individuals above 5 years old, but this was not statistically significant. In contrary with this, the result of another study shows four-fold increase in the parasite rate in children aged 2-5 years of age compared to those above 5 years [33,34].

The presence of infection among infants and children younger than 5 years old in stable areas implies autochthonous malaria transmission in the study area. That is, conventionally in areas of high endemicity, prevalence of malaria infection is known to peak at an early age with an increase up to the age of 5 years; followed by a sharp fall in age groups 10-15 years and continuing on a slow decline with increasing age (WHO, 2000 unpublished document). This pattern of prevalence is a reflection of the age-related state of anti-malaria immunity that is developed as a result of repeated malaria infections under established malaria endemicity. Studies reported that individuals living in areas of unstable and low intensity malaria transmission do not acquire significant immunity to the disease, and hence malaria infections can be observed in all age groups [35,36].

Recent work on the age-specific risk for malaria in eastern Sudan showed that the prevalence of malaria was high up to the age of 19 years [35]. Similarly, studies conducted in areas of lower malaria endemicity, for example, in Riboque in Sao Tome, had shown little or no influence of age on infection complexity [37]. Thus, the epidemiological condition prevailing in Jimma town from a prospective parasitological survey point of view suggests that the area is characteristic of an unstable, low level of malaria endemicity.

The transmission of malaria is determined by main factors such as human behavior and the existence of malaria parasite, as well as social and health facility factors such as housing condition, occupation, KAPs of the community towards malaria causation, transmission, treatment seeking behaviour and presence of mosquito control activities can affect malaria prevalence [27,28]. Importantly, a high proportion of the urban population at any age is at risk of malaria due to lack of acquired immunity [38].

The findings of this study indicated that general awareness about malaria was high among Jimma urban communities of the study site. It was considered as the major health problem in the community. About 71.8% of the study participants were aware of the fact that Plasmodium is the causes of malaria. Of the 291 visited households, about 59.5% of the respondents associated malaria to the mosquito bite and this is different from a study conducted in rural Ethiopia [39] in which 63.4% of respondents associated mosquito bite to malaria. This result was also relatively low when compared to other African courtiers, Uganda (77.6%) [40] and Kampala (84%) [41], of the respondents interviewed knew that mosquitoes transmitted malaria. The differences might be attributed to various factors. It has been considered that malaria is exclusively affecting the rural communities and as a result focused malaria control strategies have been in place which could also be the reason for the better awareness of the rural community toward mechanisms of malaria transmission better than communities of the urban areas where little &/or no strategy is place for same purposes.

Examples of misconception about causes of malaria are reported in research from all over the globe [39]. Similarly, this study showed that some community members still have misconceptions about causes of malaria. These are the major socio-cultural setbacks in malaria treatment and control. All these add up to the discrepancies in health seeking behavior and may cause delay in seeking appropriate treatment. Knowledge of the respondents about whether or not malaria is a treatable disease was significant among Jimma Town community. The results of this study have shown that about 88% percent survey respondents replied 'yes '. This appeared comparable to the rural malaria study in Ethiopia in which about 88.1% respondents replied 'yes' for the similar question [39].

Also in Jimma town potential mosquito breeding sites, comprising small, temporary, freshwater pools (man-made or natural) that are exposed to sunlight, abound in Jimma town quarters in which malaria is endemic. More breeding sites are created by human manipulation of the environment, mostly for necessary endeavours, such as housing, expansion of university and building dams for fishing. Other factors that have a direct impact on breeding sites include house structure and rubbish disposal. Overall, the absence of integrated waste management system might partially contribute to the persistence of malaria menace in Jimma town [27].

From environmental factors, only the presence of stagnant water in close proximity to house (<1 km) has shown a significant association. Studies also witness that the relationship between malaria vector density and the distance of settlement from a water body like river is an important indicator of malaria transmission, as revealed in ITNs study in Gambia, they found out an inverse relationship between the number of mosquitoes in village and the distance of settlement from the river [42]. The report of the majority of household heads in the KAP survey that they have been using anti-malaria drugs mainly chloroquine as self-treatment is typical of the situation in rural Africa where self-treatment is the most common practice in malaria. Thus, since self-treatment is most often known be associated with an improper use of anti-malaria drugs [22], the danger of spread of artemether-lumefantrine or chloroquine-resistant malaria is eminent unless measures are taken by responsible body.

Although indoor residual spray (IRS) of households has been practiced twice yearly (information obtained from the Jimma town malaria prevention and control office), the effect of this spray in stopping transmission of malaria in the area was not successful. This could be an indication of either insecticide resistance of mosquito vectors or a reflection of the inefficiency of the control measures, including human interference with the indoor residual sprays and insufficient coverage of the spray. Other factors like drug resistance may also have contributed to the lack of impact of the intervention on the transmission. This study tried to assess attitudes, knowledge and perceptions of the community regarded as urban settlers and indeed backed by actual parasitological survey of clinical and asymptomatic malaria prevalence in the study area. But this is not without certain limitation as to failure to address the issue of drug resistant, pesticide resistant and other climatic and environmental factors that can contribute the occurrence of urban malaria.

## Conclusion

Despite presence of ITNs in household was high, malaria is still a major health problem and *P. vivax *was the predominant *Plasmodium *species in the town. The prevalence was strongly associated with proximity of residence to potential mosquito breeding sites which indicates human activity plays a major role in urban malaria ranging from creating breeding sites, 'importing' cases, or through treatment-seeking choices. Solutions there¬fore, must also focus on human behavior. From different factors assessed the presence of stagnant water nearby home (<1 km) and not using of ITNs at home during sleeping were significant risk factors for malaria transmission in the town. So, in areas where the option of environmental manipulations may be difficult especially after the major rainy season, it is advisable to apply IRS. Also proper awareness creation for appropriate utilisation of ITNs and community mobilisation for environmental manipulation is crucial to minimise morbidity and mortality of malaria in the town.

## Competing interests

The authors declare that they have no competing interests.

## Authors' contributions

AA conceived the study, undertook statistical analysis and drafted the manuscript. WT, LG and GA initiated the study and made major contributions to the study design and statistical analysis. All authors contributed to the writing of the manuscript and approved the submitted version of the manuscript.
